# Between memory and oblivion: the complex experiences of ZAKA volunteers during the Israel-Hamas war (“Swords of Iron”)

**DOI:** 10.3389/fpsyg.2026.1721764

**Published:** 2026-02-11

**Authors:** Oren Cohen Cohen Zada

**Affiliations:** 1 Ono Academic College, Kiryat Ono, Israel; 2 Talpiot Academic College of Education, Holon, Israel

**Keywords:** memory and oblivion, moral injury, post-traumatic growth, trauma, trauma processing, ZAKA volunteers

## Abstract

**Introduction:**

This qualitative study characterizes the moral-psychological and existential dynamics of 24 ZAKA volunteers during the “Iron Swords” war. It focuses on how the encounter between extreme trauma and a supreme moral-Halakhic mandate (“Chesed Shel Emet”) shapes trauma processing.

**Methods:**

Through in-depth semi-structured interviews, the research examined the volunteers’ experiences of the October 7, 2023, events, their emotional difficulties, and the tension between memory and forgetting.

**Results:**

Four core themes were identified: sensory and emotional overload leading to internal conflict, the role of social support as a pillar for resilience, the “tightrope walk” between memory as a mission and oblivion as a coping mechanism, and the significance of commemoration as a source for Post-Traumatic Growth (PTG).

**Discussion:**

The findings indicate that the Halakhic commitment functions as a unique cultural-religious mediating variable. While it intensifies the risk of Moral Injury due to helplessness, it simultaneously serves as a stable collective meaning anchor that drives a resilient Collective PTG Identity. This study offers critical clinical and ethical insights for emergency organizations.

## Introduction

ZAKA (Identification of Disaster Victims) is a unique voluntary body specializing in the sensitive handling, identification, collection, and transfer of remains in situations of mass casualty events, disasters, and terrorist attacks. This activity requires exceptional technical skill and psychological resilience from its members, as volunteers are often the first to arrive at scenes of extreme human destruction, operating under immediate life-threatening danger.

Most ZAKA volunteers come from the Orthodox (Haredi) community. Their activity is driven by a deep commitment to Jewish law (Halakha) regarding death and burial. Specifically, the principle of “Chesed Shel Emet” (True Kindness) guides their work, imbuing grueling effort with profound religious and moral meaning without expectation of reward. This moral imperative elevates volunteer work from a civic duty to the status of a Supreme Moral Mandate, placing the organization at a unique intersection of religious values, civil volunteering, and national resilience.

The harrowing events of October 7, 2023, and the subsequent Israel-Hamas war (“Iron Swords”), placed ZAKA volunteers at the absolute forefront of the human tragedy (State Comptroller, 2025; Yafe et al., 2019). Volunteers operated in extensive, prolonged, and extremely dangerous scenes, experiencing direct exposure to loss and horrific sights and smells. This unprecedented level of exposure, combined with religious duty, created a unique crucible for psychological and moral distress.

While existing literature has examined trauma among first responders, a critical research gap remains in analyzing the personal and moral-psychological experiences of volunteer first responders operating under a supreme moral mandate. Standard trauma models often fail to explain the unique stress mechanisms at the intersection of religious duty and human limitation.

This study seeks to bridge this gap through an in-depth examination of the moral-psychological and existential dynamics characterizing the unique encounter between extreme trauma exposure and a supreme moral-Halakhic mandate among 24 ZAKA volunteers who operated at the combat scenes on October 7, 2023.

The primary objectives of this research are:

- To characterize the moral-psychological and existential experience of ZAKA volunteers, examining how the conflict between absolute religious duty and overwhelming traumatic reality affects trauma processing and the tension between memory and oblivion.
- To understand the emotional and psychological difficulties faced by ZAKA volunteers, with a specific focus on the tension between memory and oblivion in the trauma processing.
- To utilize the volunteers’ narratives to expand theoretical models of Moral Injury (MI) and Post-Traumatic Growth (PTG).

The central theoretical contribution of this study is the placement of moral-Halakhic commitment (“Chesed Shel Emet”) as the central analytical lens: a mediating variable that simultaneously generates a high risk for endogenous Moral Injury (MI) due to the “Impossible Moral Mandate” and functions as a stable Collective Meaning Anchor for post-traumatic growth. This research provides vital insights for developing culturally-adapted interventions for similar emergency organizations worldwide driven by a moral mandate.

## Theoretical background

This literature review examines the psychological and social consequences of repeated trauma exposure among volunteers in disaster zones, focusing on the activity of ZAKA volunteers in extreme situations. The review relies on Israeli and international studies, highlighting their relevance to ZAKA volunteers in Israel.

### Trauma exposure and its intensity among ZAKA volunteers

ZAKA volunteers, who operate in the traumatic scenes of terror attacks, natural disasters, and severe accidents, are exposed to high levels of trauma. Many studies indicate that direct exposure to scenes of violence, horror, and severe physical injuries creates an increased risk for psychological disorders, primarily Post-Traumatic Stress Disorder (PTSD), anxiety, depression, and feelings of guilt. In their work, volunteers are required to cope with witnessing mutilated bodies (Yoshida et al., 2023; Manhart et al., 2012), sometimes under time pressure and physical pain, as well as security risks in conflict zones (Boer et al., 2020). Numerous studies show that exposure to dead bodies-during identification, evacuation, or burial processes-can cause psychological distress (Avital, 2024; Ben-Ezra et al., 2006). Exposure to death constitutes a risk factor for PTSD (Solomon et al., 2007).

### Psychological and personal coping mechanisms

As part of coping with trauma exposure, ZAKA volunteers employ psychological defense mechanisms such as suppression, denial, emotional detachment, and the creation of a sense of control. Conversely, the long-term repression of traumatic emotions can lead to symptomatic exacerbation, negatively affecting quality of life and daily functioning (Yildiz et al., 2023; Meredith et al., 2011). One of the notable characteristics among volunteers is the lack of seeking psychological treatment. One of the notable characteristics among emergency service personnel is the lack of seeking psychological treatment. Barriers such as self-resilience, organizational culture, fear of occupational consequences, and social stigma prevent them from opening up and receiving professional assistance (Auth et al., 2022). Additionally, there is sometimes an organizational approach that encourages “resilience” and bearing the burden alone, instead of promoting a culture of psychological support (Zelenska, 2021).

### The theoretical dimension in trauma research: moral injury and post-traumatic growth

Current research identifies two additional central response mechanisms, essential for understanding the volunteers’ experience: Moral Injury (MI) and Post-Traumatic Growth (PTG). Moral Injury (MI) is defined as a profound violation of a person’s basic moral expectations following an act, omission, or witnessing of an event that violates strong moral and ethical values (Litz et al., 2009). Unlike PTSD, which stems from fear and bodily danger, MI leads to feelings of guilt, shame, anger towards the self and the world, and difficulty reintegrating into the community. For first responders, MI often arises when they are forced to make difficult choices, or when organizational reality and limited resources prevent them from fully fulfilling their moral duty (Griffin et al., 2019). The current study will examine how the religious-Halakhic commitment of “Chesed Shel Emet” may amplify the unbridgeable gap between the moral demand for action and human and practical limitations, thereby constituting a risk factor for MI. PTG describes positive psychological changes that occur as a result of coping with a significant traumatic event, which are not merely a return to the previous state, but an improvement upon it (Tedeschi and Calhoun, 1996). For emergency volunteers, finding meaning through the activity (commemoration, assistance) is the central pathway to achieving PTG. The current study will examine how ZAKA’s religious-Halakhic and communal framework serves as a unique anchor for the PTG process, as it immediately provides a “collective meaning framework” that strengthens individual and group resilience.

### Memory, oblivion, and emotional processing

Trauma involves a complex combination of painful memory and necessary oblivion, aimed at protecting the psyche from unbearable emotional overload. ZAKA volunteers cope with the difficulty of preserving a personal and collective memory of the traumatic events-through documentation, testimonies, and commemoration-alongside the need for partial oblivion that allows them to continue functioning under continuous stress (Newman et al., 2024). Studies indicate that the right to remember and forget is a key component in maintaining mental health and coping with trauma. The freedom to choose which memories to hold and which to dismiss is a crucial psychological defense tool, which prevents emotional flooding and allows for an internal healing process. Volunteers in organizations like ZAKA are under constant tension between the need to preserve the memory of the victims, for commemoration ceremonies, and for a sense of personal and social obligation, and the need to forget part of the experience in order to maintain their functioning in the field and in their personal lives (Howes et al., 2023; Boer et al., 2020). Therefore, understanding these dynamics is a vital key to developing effective and culturally adapted psychological support for ZAKA volunteers in extreme situations (Solomon and Berger, 2005).

### The Halakhic commitment: a unique protective factor versus a risk factor

The unique context of ZAKA volunteers’ activity stems from the Halakhic commitment to “Chesed Shel Emet” (True Kindness). This commitment serves as a primary protective factor by providing a deep religious meaning that helps the volunteers cope with the trauma. At the same time, this very Halakhic injunction constitutes a unique risk factor: the strict duty to collect every human remnant for burial necessitates prolonged and excessive active presence in the most traumatic scenes, thereby potentially preventing defensive psychological withdrawal (Zarfati, 2022). This unique tension, between the internal Halakhic mandate and the prolonged external exposure, is the core theoretical contribution of this study within the religious-cultural context.

To conclude this literature review, ZAKA volunteers during the October 7, 2023, events coped with extreme psychological and physical overload, exposed to severe and sometimes incomprehensible trauma. They employed diverse psychological defense mechanisms, including repression and denial, and required significant social and organizational support. Processes of memory and oblivion were central to their emotional coping, alongside significant consequences for their personal lives.

## Methodology

### Research approach and rationale

This study is grounded in a qualitative-phenomenological paradigm, aimed at uncovering and meticulously analyzing the subjectivity of experience and the profound processes of meaning-making from the perspective of ZAKA volunteers who operated during the “Iron Swords” war. This methodology is essential for an in-depth exploration of the Experiential Focus in the current study, which is situated at the existential intersection of an absolute moral-religious mandate and exposure to extreme trauma. This choice enables the decoding of the moral-psychological dynamics underlying the structural tension between Moral Injury (MI) and Post-Traumatic Growth (PTG) (Given, 2021; Creswell and Poth, 2018).

By employing this approach, we aim to distill rich phenomenological insights into how the “Supreme Moral Mandate” of “Chesed Shel Emet” (True Kindness) simultaneously triggers both the “Impossible Moral Mandate” (the source of MI) and the formation of a “Collective PTG Identity.” These are complex, multi-layered narrative processes that are not accessible through standard quantitative measures.

### Study population and sample

The study utilized semi-structured in-depth interviews, recognized as the most suitable method for eliciting rich data concerning motivations, perceptions, complex emotions, and adaptive coping mechanisms (Patton, 2022) within the context of high-stress humanitarian response.

The interview protocol development process was anchored in a thorough literature review, followed by a strategic four-person pilot study. The pilot was crucial not only for testing the clarity and emotional sensitivity of the questions (Johnson and Turner, 2021) but, more importantly, for ensuring the protocol was culturally and ethically sensitive to elicit meaning-making narratives related to the moral-religious conflict. The four pilot volunteers represented the diversity of roles, tenure, and ages. Following the pilot, slight adjustments were made to the phrasing and order of questions, enabling more fluent, ethically informed, and targeted interviews focused on the core themes of moral conflict and psychological transformation (i.e., PTG).

The role of ZAKA volunteers was significantly expanded and altered during the “Swords of Iron” war due to the exceptional circumstances on the combat ground. Beyond their known core missions of body collection and identification, volunteers were required to perform a wide range of additional roles, including: logistics and field coordination, documentation for legal purposes, rabbinic guidance on Halakhic (Jewish law) matters, psychological support for security forces and other volunteers, and operations in sites not yet fully secured. This expansion was necessitated by high operational demands, security risks, logistical challenges, and the need for holistic support.

The following table, Table 1, summarizes the variety of roles performed by ZAKA volunteers in the field, based on in-depth interviews conducted with them (*N* = 24).

**TABLE 1:** Roles of ZAKA volunteers in the “Swords of Iron” war (*N* = 24).

ZAKA role in the war	Percentage of volunteers who performed the task	Defined special challenges
Body collection	85%	Dealing with dangerous scenes, bodies in states of severe burning.
Psychological support	80%	Providing initial emotional support to police, soldiers, and other volunteers.
Body identification	75%	Time pressure, bodies in states of fragmentation or decomposition.
Contact with bereaved families	60%	Delivering traumatic information, emotional accompaniment.
Logistics and field coordination	55%	Equipment management, coordination between various bodies.
Documentation and photography	50%	Documenting evidence and photography at the scenes for legal identification.
Rabbinic guidance/Halakhic matters	40%	On-the-ground Halakhic (Jewish law) decisions.
Transport and transfer	35%	Transferring bodies and equipment in dangerous areas.
Assistance in unsecured scenes	25%	Working under life-threatening risk, scenes with heavy fire.
Liaison with forensic institutions	10%	Coordination and transfer of bodies to hospitals, morgues, and the Forensic Institute.

### Research tools

The primary research tool was a semi-structured in-depth interview, which allowed for the collection of rich narrative data while maintaining consistency alongside the flexibility required to explore emerging themes of trauma and resilience (Johnson and Turner, 2021; Turner, 2020). Participants were recruited using the Snowball Sampling method from within the community. The interviews were conducted in person in locations that provided full privacy and quiet—most often in the participants’ homes or private offices of their choice—to facilitate open discussion on sensitive topics.

Data collection continued until Theoretical Saturation was achieved—meaning that no substantial new information was received regarding the central theoretical concepts (such as the “Impossible Moral Mandate” and “Collective PTG Identity”). This process was executed through Constant Comparison between the interviews. Saturation was reached by the 21st interview, at which point data analysis yielded no further new concepts or themes related to the moral mandate or growth. The remaining three interviews (22–24) were conducted to validate the findings and ensure that no exceptional experiences remained unexpressed.

During the interviews, participants were guided by core questions designed to elicit data on trauma exposure, moral conflict, and psychological transformation. Key questions included:

- How did the volunteers experience their activity in ZAKA, and what were the most challenging moments?
- What personal, religious, and communal meanings are attributed to this activity, and how did these meanings drive psychological change or growth?
- What difficulties and challenges did they encounter?
- How did the activity affect their personal lives, and what mechanisms enabled successful coping?
- How did the tension between memory and oblivion manifest in their emotional processing (reflecting the ongoing struggle for resilience)?

### Data analysis

The interview transcripts were analyzed using the Thematic Analysis method, based on open inductive coding where recurring concepts and topics (codes) were identified and grouped into core themes (Nowell et al., 2017). The analysis maintained a critical balance between inductive openness and reference to the established theoretical and cultural contexts, focusing specifically on identifying patterns of meaning-making, moral conflict, and evidence of post-traumatic growth and resilience (Smith and Firth, 2023).

Trustworthiness was strengthened by adhering to four key criteria (Lincoln and Guba, 1985), ensuring the scientific rigor of the qualitative findings:

Credibility: Achieved through Member Checking-selected participants received a summary of their core narratives and the central themes, and were given the opportunity to correct or add information (Vella, 2024).

- Dependability: Achieved through Co-coding-representative transcripts (20% of the total interviews) were coded by an expert colleague researcher, achieving an inter-coder agreement of 85%. Discrepancies were resolved through mutual discussion.
- Transferability: Achieved through Thick Description-a detailed and rich description of the organizational, religious, and cultural context (the *Supreme Moral Mandate* context), as well as detailed demographic data (Table 2), was maintained, allowing other researchers to evaluate the relevance of the findings to similar morally-driven populations.
- Confirmability: Achieved through Process Transparency-recordings, transcripts, and a Decision Trail (Audit Trail) were preserved to allow for traceability and oversight throughout the process.

**TABLE 2:** Demographic and occupational characteristics of study participants—ZAKA volunteers in the Israel-Hamas war (*N* = 24).

Variable	Category	Number of participants	Percentage of participants
Area of residence	North	6	25%
	Center	3	12.50%
	South	15	62.50%
Age	35–39	6	25%
	40–45	16	66.70%
	51 and above	2	8.30%
ZAKA tenure	2–4 years	2	8.30%
	5–7 years	6	25%
	8 years and above	16	66.70%
Marital status	Married	20	84%
	Widowed	4	16%
Primary occupation	Paramedic	6	25%
	Nurse/caregiver	5	20.80%
	Volunteer team manager	4	16.70%
	Firefighting and rescue	3	12.50%
	Other	6	25%
Education	High school	12	50%
	Academic	9	37.50%
	Religious studies	3	12.50%
Religiosity level	Religious/ultra-orthodox	24	100%
Direct exposure at scene	Yes	24	100%
Prior combat operations	Yes	24	100%

To ensure full methodological transparency and compliance with international standards for reporting qualitative research, Table 3 details the study’s adherence to the COREQ criteria:

**TABLE 3:** Adherence to COREQ criteria.

COREQ item	Implementation details in the study
Research team	The principal investigator is a trauma-specialist psychotherapist with deep familiarity with the religious/Haredi culture.
Sampling method	Purposeful sampling combined with the “Snowball” sampling method.
Interview location	Private and quiet locations (participants’ homes or offices) to ensure comfort and discretion.
Data collection	Semi-structured in-depth interviews (60–90 min), fully recorded and transcribed.
Saturation	Saturation was achieved by the 21st interview; three additional interviews were conducted to confirm the findings.

### Research ethics

Participation in the study was voluntary, including a detailed explanation of the research objectives, the work process, the right to refuse or withdraw at any time without consequences, and the receipt of signed informed consent (Johnson and Turner, 2021). The research protocol and procedures were fully approved by the Institutional Review Board (IRB) prior to the commencement of data collection.

Given the study’s focus on a highly sensitive group exposed to extreme trauma, maintaining full discretion and high ethical sensitivity was a central element of the data collection. The ethical procedures were specifically designed to safeguard the well-being of the participants in trauma research, a critical requirement for this journal (Nowell et al., 2017).

All personal and professional information was handled anonymously, and interview transcripts were scrubbed of identifying details and used pseudonyms to protect participant privacy. The researcher maintained cultural congruence by addressing the religious and social values of the participants to create a safe, respectful environment for the expression of emotions. Crucially, the researcher was trained in identifying signs of psychological distress and providing psychological first aid. In cases where distress signs were observed during the interviews, the interview was immediately stopped, and initial emotional support was provided.

Furthermore, the current study was conducted independently and not in official partnership with ZAKA, and the participants’ involvement was not reported to the organization to protect their autonomy. The entire process was conducted in strict adherence to principles of research ethics, emphasizing cultural and psychological sensitivity, and maximal protection of participant privacy, ensuring an ethical, reliable, and respectful research process (Creswell and Poth, 2018).

## Findings

This research uncovered four central and sequential themes, reflecting the complex experiences of ZAKA volunteers in the process of emotional processing and meaning-making following extreme trauma. These themes outline the spectrum between vulnerability (risk of Moral Injury—MI) and Post-Traumatic Growth (PTG). To enhance analytical transparency and facilitate an integrative understanding of the findings, Table 4 provides a hierarchical mapping of the themes revealed in the study. The table presents the dynamic relationship between stressors and moral conflict, and the resilience and growth resources among the volunteers.

**TABLE 4:** Mapping of themes and sub-themes—the moral-psychological dynamics in the experience of ZAKA volunteers in the “Iron Swords” war.

Central theme	Sub-themes and experiential components	Interrelations and analytical dynamics
1. The impossible moral mandate	∙Extreme sensory and emotional overload ∙Flashbacks and intrusive memories ∙Conflict between Halakhic duty and human helplessness	Constitutes the basis for the development of Moral Injury (MI) due to the gap between the absolute command and the reality of destruction.
2. Social support as a basis for resilience	∙Reduction of loneliness through shared fate ∙Deep understanding within the peer group ∙Non-judgmental listening as a “lifeline”	Serves as a protective mechanism that mitigates Moral Injury and enables the transition from a private world to a collective narrative.
3. The tension between memory and oblivion	∙Memory as a moral mission and loyalty to the deceased ∙Oblivion as a survival necessity for functioning ∙Creation of psychological “balancing mechanisms”	A direct expression of the conflict in Theme 1; the need to walk a “tightrope” between preserving identity as a witness and psychological recovery.
4. Meaning anchor for growth (PTG)	∙Finding meaning within suffering ∙Activity and commemoration as tools for processing and closure ∙Formation of a “Collective PTG Identity”	The commitment to “Chesed Shel Emet” transforms from a risk factor (Theme 1) into a source of strength and growth anchored in permanent religious values.

To enhance the contextual richness of the findings, limited socio-demographic and professional data of the participants are presented alongside the selected quotes. This information (such as age group, profession, and organizational tenure) is based on the data detailed in the Methodology section (see Table 2). The data are presented in ranges and in a manner that precludes personal identification, ensuring the strict confidentiality and anonymity of the participants in accordance with ethical protocols.

### Theme 1: the impossible moral mandate: exposure, overload, and the genesis of moral injury

This theme illustrates that the volunteers’ exposure to severe traumatic scenes created a sustained emotional and sensory overload, serving as the backdrop for the development of an Endogenous Moral Conflict between the sense of supreme commitment and the feeling of human limitation and helplessness. This conflict distinctly represents the potential for Moral Injury (MI).

The exposure was described as an overwhelming process that created continuous primary trauma, manifesting as intense emotional strain that persisted long after the activity ended, including intrusive memories, flashbacks, and physical sensations of tension and anxiety.

R.B (Age: 43, Paramedic, Tenure: 12 years): “The pain I felt when I entered the scene was so deep. I saw bodies scattered next to burnt houses, and the feeling was one of inner collapse. When I got home, I couldn’t shake the memories, as if they accompany me constantly. Every truck that passes on the road triggers a flashback of trucks carrying bodies.”
Sh.B (Age: 37, Nurse, Tenure: 6 years): “The smells and sounds of the scene are etched in my memory. Sometimes at night, I wake up from the memories, as if I am there again, in the center of the pain and loss. The experience wasn’t just visual, it was physical and emotional pain simultaneously.”

Crucially, this physical and emotional overload directly clashed with the deeply ingrained sense of absolute Halakhic commitment to act for the dignity of the deceased (“Chesed Shel Emet”). The volunteers experienced a paralyzing conflict between this moral-religious imperative (representing the commitment) and the feeling of despair, heaviness, and helplessness in the face of the disaster’s magnitude and the limits of personal capacity.

**R.F** (Age: 54, Volunteer Team Manager, Tenure: 15 years): “I felt a true obligation to be there, to help with everything I could. However, I often felt that we were failing against the scale of the disaster. Every time I arrived at a scene, in the first moments it was clear to me that we had to act. But inside, deep in my soul, I felt as if I was being swallowed by an abyss.”
**R.Tz.** (Age: 41, Firefighting and Rescue, Tenure: 9 years): “Within that feeling of mission, a paralyzing sense of helplessness repeatedly crept in. Sometimes, after finishing at the scene, I had to stop, breathe deeply because the soul hurt more than the body.”

This difficult duality-an absolute moral commitment colliding with an inevitable sense of failure-reinforces the core tension of the Impossible Moral Mandate, setting the stage for the analysis of MI as an endogenous phenomenon stemming from the violation of a sacred duty.

### Theme 2: social support as the foundation for collective resilience

In contrast to the moral and emotional distress described in Theme 1, the volunteers universally highlighted the critical importance of mutual support and interpersonal connections forged within the teams. This support system served as a powerful protective mechanism (*psychological resource*) that strengthened resilience and enabled the shared processing of traumatic experiences. This internal social system was perceived as the primary source of collective strength.

R.Tz. (Age: 41, Firefighting and Rescue, Tenure: 9 years): “We felt we were not alone in the storm. Within the severe pain, I found a listening ear and a shoulder to lean on. It was truly like a refuge that made me feel I could continue.”
M.Sh. (Age: 44, Paramedic, Tenure: 11 years): “When I talk to my teammates, I feel we understand each other in a deep way that isn’t possible elsewhere. This gives me a feeling of belonging and trust.”
M.L. (Age: 38, Freelance Professional, Tenure: 3 years): “The support I felt from the group of volunteers who stood by my side in those unimaginable scenes, who saw with me what a human eye is not supposed to see, was like a lifeline.”

In summary, these findings illustrate that the deep social connections within the volunteer teams transcended basic support; they served as the necessary infrastructure for building collective resilience, providing a shared narrative of commitment that countered the individual feeling of moral failure.

### Theme 3: the tightrope walk: memory as a moral mission vs. oblivion as a coping mechanism

This theme describes the volunteers’ deep emotional conflict between the moral obligation to commemorate and preserve the memory (out of commitment and loyalty to the victims) and the profound human need for oblivion or detachment to maintain mental health. The tension between memory and oblivion was a central challenge in their emotional processing.

R.F. (Age: 54, Volunteer Team Manager, Tenure: 15 years): “I feel committed to keeping the memory alive and strong, not just for me, but for them too. For those who didn’t get to be remembered in another way. Every action I take. is part of the effort to commemorate. But often, the pain becomes unbearable. There is a strong need in me to run away, not to forget, but to breathe.”
L.M. (Age: 42, Nurse, Tenure: 10 years): “Oblivion is tempting. It promises quiet, a promise of routine and normal life. But at the same time, it feels like a betrayal, as if in the very surrender of memory, I sin against the truth. Every thought of moving away from the pain is accompanied by pangs of conscience.”

The volunteers perceive themselves as “Memory Keepers”-loyal to the victims and the traumatic experience. For them, memory is not a choice, but a sacred mission, yet it becomes a chronic psychological burden.

Testimonies indicate that, to cope, many had to construct “balancing mechanisms”-ways to distance the memory without erasing it, transforming the narrative to enable sustained functioning. Here, the strength of the supportive community (Theme 2) re-emerges, allowing the transformation of memory from a debilitating private burden into a communal subject for processing, significantly reducing the individual psychological load and enhancing resilience.

### Theme 4: the collective meaning anchor: activity and commemoration driving PTG identity

The final theme highlights how the continuation of volunteer activity and involvement in commemoration ceremonies served as profound meaning-making experiences, providing a sense of mission, belonging, and inner strength-core characteristics of Post-Traumatic Growth (PTG). The activity transcended immediate response; it became a significant processing tool that facilitated finding meaning within the pain.

B.C. (Age: 52, Volunteer Team Manager, Tenure: 14 years): “Participating in ceremonies, lighting candles, and remembering the stories of the fallen helped me find meaning, to feel that we are not just coping but also remembering and acknowledging.”
L.Sh. (Age: 36, Freelance Professional, Tenure: 5 years): “I see how the community embraces and honors the victims, and that gives me strength to continue contributing. It strengthens me and fills me with meaning.”
R.Tz. (Age: 41, Firefighting and Rescue, Tenure: 9 years): “Volunteer activity and commemoration are tools that allow me to close a circle, to feel that the pain is part of something bigger, and that we are not alone.”

The meaning attributed to the activity is a key factor in trauma processing and achieving psychological resilience. Crucially, this meaning is anchored in the Halakhic motivation of “Chesed Shel Emet.” This religious-moral commitment provides a fixed, supreme value system that allows for the creation of a renewed, meaningful self-narrative, leading not just to individual PTG, but to a Collective PTG Identity-a shared sense of growth and resilience rooted in a higher moral calling. This collective narrative is key to their long-term persistence and functioning capability.

## Discussion

The findings of this qualitative study reveal the moral-psychological and existential dynamics inherent in the work of first responders operating under “Supreme Moral Mandates,” specifically ZAKA volunteers following the horrific events of October 7, 2023. The four central themes not only align with existing trauma research but, crucially, offer a significant theoretical expansion to prevailing models of Moral Injury (MI) and Post-Traumatic Growth (PTG) (Byrd and Ross, 2024; Jones and Majied, 2009).

### Expanding the moral injury model: the impossible moral mandate

The first theme, describing the intensity of direct exposure and ongoing emotional burden, is consistent with literature linking severe trauma exposure to prolonged distress and symptoms of PTSD (Yildiz et al., 2023). Crucially, this ongoing emotional load, when coupled with a deep religious commitment, generates the central conflict identified here: the clash between the volunteers’ “Absolute Moral Mandate” (the sacred duty) and the inevitable helplessness faced against the overwhelming scale of the disaster.

This conflict clearly represents an innovative and endogenous (internal) form of moral injury. Unlike prevailing MI models, which typically focus on injury stemming from a “betrayal” by leadership or the commission of a transgressive act that violates an external moral code (Litz et al., 2009), the current findings suggest that for ZAKA volunteers, the injury stems precisely from the violation of a deep internal moral expectation—the “Impossible Moral Mandate.” This insight expands the integrative review of Griffin et al. (2019) and proposes that in mission-driven communities, the source of injury is internal and existential, anchored in faith, rather than merely organizational or operational.

### Collective meaning, resilience, and collective PTG identity

The second and fourth themes demonstrate the unique paradoxical function of this supreme moral commitment: while it generates a risk for internal MI, the shared duty simultaneously serves as a powerful collective meaning anchor that drives resilience and growth. The second theme corresponds with resilience studies among emergency professionals in disaster zones (Nissen et al., 2022).

However, the current study challenges the traditional conception of PTG as an individual transformation process within the individual’s belief system (Tedeschi and Calhoun, 1996). We propose the concept of “Collective PTG Identity,” which embodies the growth of the entire group around shared religious values. Thus, the research suggests a paradigmatic shift from a focus on individual-fragmented PTG toward a model of systemic-communal growth, where the shared identity acts as a highly resilient buffer against future stress challenges (Nissen et al., 2022).

### The ethical challenge: memory, oblivion, and the path to recovery

The third theme highlights the tension between the moral commitment to memory and the psychological need for oblivion to preserve functioning. This chronic tension reveals a deeper conflictual layer than those described in the literature concerning Body Handlers. While studies by Boer et al. (2020) and Howes et al. (2023) focus on the technical and emotional challenges of Disaster Victim Identification (DVI), the current study shows how these transform into an ethico-moral conflict due to the absolute Halakhic duty. These findings confirm and expand the early findings of Solomon et al. (2007) regarding the repressive coping style of ZAKA personnel, suggesting that psychological “balancing mechanisms” are essential for mental recovery in the face of the Supreme Moral Mandate.

The unique structure of endogenous MI and collective PTG observed here necessitates culturally-adapted clinical interventions:

- Reframing Moral Failure as Moral Heroism: Interventions must focus on the validation of the moral injury, but strategically, they should reframe the “failure to fully fulfill the mandate” as an act of moral heroism performed within an impossible situation. The emphasis should shift from the inevitable lack to the supreme sacrifice made in the attempt to honor the sacred duty.
- Strengthening Collective PTG Identity: Systemic support must leverage the power of the group’s shared purpose. We recommend strengthening post-event social-religious forums that validate the shared mission and reinforce the bonds of the “moral community” to consolidate their collective PTG identity as a primary protective factor.

**FIGURE 1:**
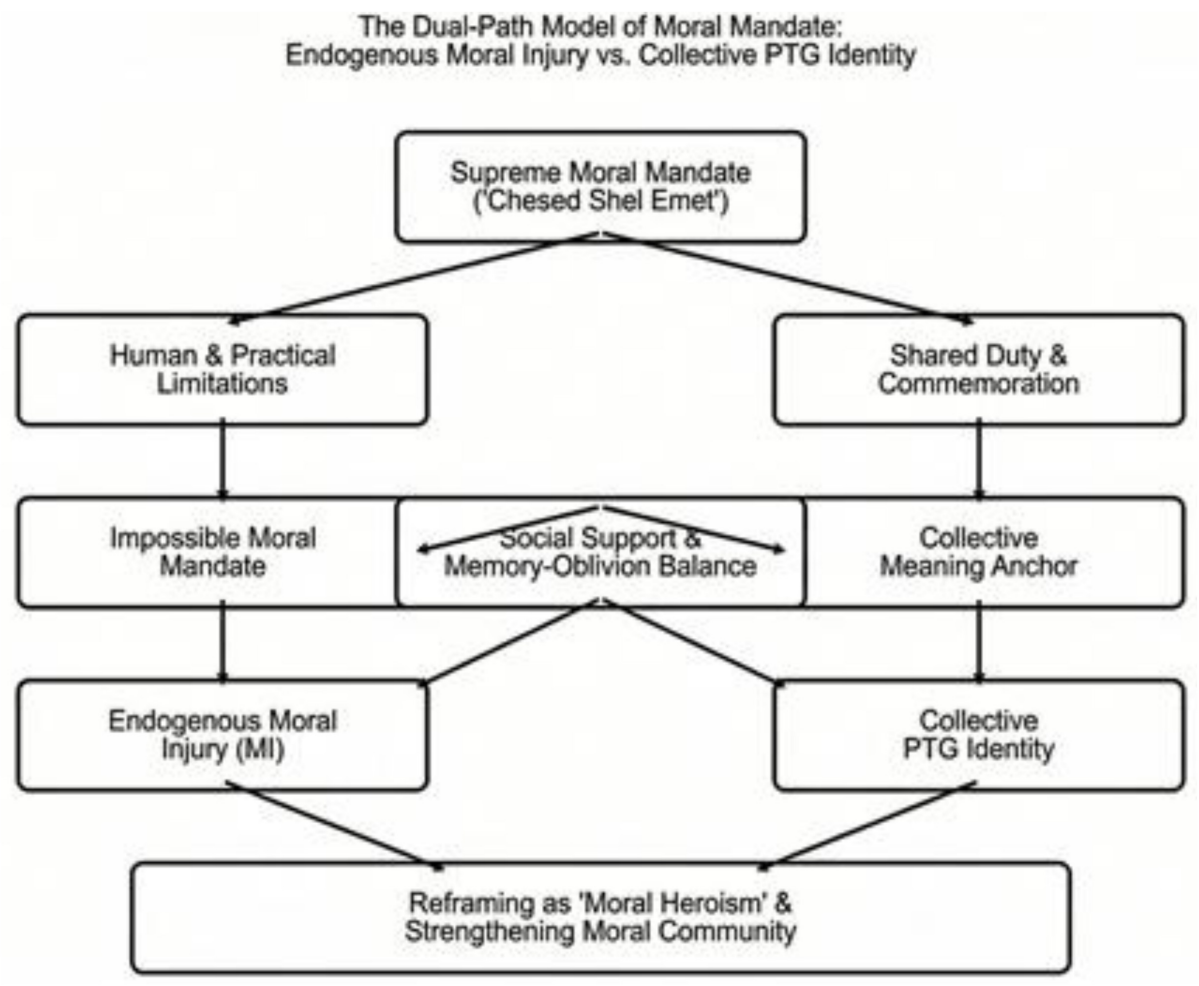
The dual path model of religious mandate in trauma exposure.

As a synthesis of the theoretical dialogue, the findings indicate that psychological safety and resilience among first responders in mass-casualty disaster zones do not depend solely on individual defense mechanisms, but on the system’s ability to provide a “moral space” for processing. While classical trauma literature tends to separate risk factors from resilience factors, the “Dual Path” model (Figure 1) emphasizes how the same value imperative—*Chesed Shel Emet*—serves simultaneously as a risk factor for MI and as the central anchor for growth. This understanding requires a transition from a symptom-management paradigm to a paradigm of identity and values integration in complex trauma treatment.

## Conclusion and Implications

Accordingly, this study provides vital insights for the development of systemic and ethically informed support protocols for any mission-driven first responder community operating under a supreme moral mandate worldwide. By introducing the concepts of the “Impossible Moral Mandate” and “Collective PTG Identity,” this research fundamentally expands the theoretical framework for understanding the interplay between trauma, moral identity, and psychological resilience.

In conclusion, this qualitative study reveals the unique psychological complexity of ZAKA volunteers during the “Iron Swords” war, emphasizing the profound tension between a supreme religious commitment (“Chesed Shel Emet”) and the imperative of trauma processing. The findings confirm the link between exposure to extreme trauma and psychological burden; however, the primary theoretical contribution lies in the fundamental expansion of the Moral Injury (MI) model.

We propose that MI in this context does not stem from a flawed command or organizational failure, but rather from an Impossible Moral Mandate—the internal, inevitable inability to fully fulfill the ultimate Halakhic duty in the face of the overwhelming magnitude of the devastation. Concurrently, the religious commitment plays a dual and paradoxical role as a stable collective meaning anchor, actively driving a more resilient form of Post-Traumatic Growth (PTG).

### Conclusion

This qualitative study reveals the unique psychological complexity of ZAKA volunteers during the Israel-Hamas war (“Swords of Iron”), highlighting the profound tension between a supreme religious commitment (“Chesed Shel Emet”) and the imperative of trauma processing. The findings confirm the link between extreme trauma exposure and psychological burden, but the primary theoretical contribution lies in fundamentally expanding the Moral Injury (MI) model.

We propose that MI in this context stems not from a faulty command or organizational failure, but from an Impossible Moral Mandate-the inevitable, internal inability to fully fulfill the ultimate Halakhic duty in the face of devastation’s overwhelming magnitude. Concurrently, the religious commitment serves a dual, paradoxical function as a stable Collective Meaning Anchor, which actively drives a more resilient form of Post-Traumatic Growth (PTG).

### Clinical and systemic implications

From these theoretical findings, focused clinical and systemic recommendations for supporting first responders operating under similar supreme moral imperatives are derived:

1. Reframing Moral Failure as Moral Heroism: Psychological interventions for morally-driven trauma must be religiously and culturally sensitive. Treatment should include the strategic reframing of the “failure of action” (the inability to collect every remnant) as an act of Moral Heroism performed within an impossible mission. Therapy must allow the volunteer to anchor their self-worth not in the operational outcome, but in the commitment’s intent and the unimaginable act performed despite all human limitations.
2. Implementing Culturally Adapted Training: Given that the dissociation observed among many volunteers stems not merely from a survival instinct but from the religious duty to concentrate (maintaining composure to fulfill the commandment), training teams should introduce “Adapted Emotional Withdrawal Mechanisms.” These mechanisms must respect Halakhic and cultural boundaries while establishing defined “mental safety spaces” that allow for conscious, contained emotional processing.
3. Solidifying Collective PTG Identity: Since Post-Traumatic Growth (PTG) arises from the group commitment to “Chesed Shel Emet,” systemic resources should be invested in strengthening the volunteers’ post-event social-religious forums. Participation in commemoration ceremonies and follow-up activities that reinforce purpose and shared mission should be highly encouraged, as this preserves their Collective PTG Identity as the primary, long-term resilience resource.

### Limitations and future research

It must be acknowledged that the present study, being qualitative research based on in-depth interviews, is limited in its ability to achieve statistical generalizability of its findings. The sample (*N* = 24) is a purposeful sample, and the narratives deeply reflect a unique religious-Halakhic and organizational background. Consequently, the direct Transferability of the findings to other emergency populations not bound by the religious imperative of “Chesed Shel Emet” is limited.

However, this unique nature constitutes the study’s tremendous strength: the findings, which surface the moral conflicts (MI) and PTG through the lens of this singular commitment, provide critical and unprecedented practical insights for mental health support systems. These insights should be immediately utilized to develop specialized, culturally-tailored intervention models for ZAKA volunteers and similar emergency organizations globally. Future research should prioritize the development and validation of quantitative instruments to measure the Impossible Moral Mandate and the Collective PTG Identity across diverse, mission-driven first-responder groups.

## Data Availability

The original contributions presented in this study are included in this article/supplementary material, further inquiries can be directed to the corresponding author.
